# Estimation of Infiltration Volumes and Rates in Seasonally Water-Filled Topographic Depressions Based on Remote-Sensing Time Series

**DOI:** 10.3390/s21217403

**Published:** 2021-11-07

**Authors:** Pavel P Fil, Alla Yu Yurova, Alexey Dobrokhotov, Daniil Kozlov

**Affiliations:** 1 V. Dokuchaev Soil Science Institute, 109017 Moscow, Russia; alla.yurova@gmail.com (A.Y.Y.); daniilkozlov@gmail.com (D.K.); 2 Department of Geography, Lomonosov Moscow State University, 119991 Moscow, Russia; 3 Agrophysical Research Institute, 195220 St. Petersburg, Russia; dobralexey@gmail.com

**Keywords:** closed depressions, temporary water bodies, remote sensing, infiltration

## Abstract

In semi-arid ecoregions of temperate zones, focused snowmelt water infiltration in topographic depressions is a key, but imperfectly understood, groundwater recharge mechanism. Routine monitoring is precluded by the abundance of depressions. We have used remote-sensing data to construct mass balances and estimate volumes of temporary ponds in the Tambov area of Russia. First, small water bodies were automatically recognized in each of a time series of high-resolution Planet Labs images taken in April and May 2021 by object-oriented supervised classification. A training set of water pixels defined in one of the latest images using a small unmanned aerial vehicle enabled high-confidence predictions of water pixels in the earlier images (Cohen’s Κ = 0.99). A digital elevation model was used to estimate the ponds’ water volumes, which decreased with time following a negative exponential equation. The power of the exponent did not systematically depend on the pond size. With adjustment for estimates of daily Penman evaporation, function-based interpolation of the water bodies’ areas and volumes allowed calculation of daily infiltration into the depression beds. The infiltration was maximal (5–40 mm/day) at onset of spring and decreased with time during the study period. Use of the spatially variable infiltration rates improved steady-state shallow groundwater simulations.

## 1. Introduction

Shallow groundwater is present in many semi-arid landscapes across the world either intermittently or permanently, depending on the lithological profile, topography, and water balance. Unlike in wetter environments with diffuse groundwater recharge, recharge in these environments is primarily focused (local) in areas of excess water input [1]. In such environments, where moisture deficits in upland soils are high, groundwater recharge will only occur if there is sufficient infiltration of converging flow to overcome the deficits. One mechanism involved is a localized recharge process that routes surface water runoff within the landscape to topographically low areas (depressions), allowing infiltration of water through ephemeral seasonal ponds [2,3,4]. Moreover, depression-focused recharge driven by snowmelt is a major annual hydrological event in cold semi-arid regions such as the Pothole Prairie Region of North America. In recent decades, there has been an accelerated increase in process understanding of the contributions of prairie potholes to surface runoff [5,6] and depression-focused groundwater recharge [3] in this part of North America. The knowledge has been acquired through studies involving conceptual and mathematical modeling of hydrological processes of surface flows [5,6,7], subsurface flows and combinations of the two [8,9], applications of isotopic and environmental tracers [3], digital elevation model (DEM)-based delineations of depressions and their watersheds [10,11,12,13,14], assessments of hydrologic connectivity [6,10,15,16,17], and remote sensing with high and intermediate resolution [18,19]. Studies in various catchments have shown that both horizontal and vertical connectivity in pothole hydrological systems are very site-specific and no model can be applied to a new system without validation. 

In Russia, areas rich in pothole-like systems of depressions (“zapadiny”) in interfluves of forest-steppe catchments cover a larger region than in North America, extending across much of European Russia and into Siberia. However, after an initial period of intensive hydrological research in the 1960s to the 1980s there was very little study of depression-focused groundwater recharge despite advances in GIS-facilitated simulation and remote sensing. Moreover, there is increasing societal need for such studies to enhance the understanding of key landscape functions related to water storage or movement, e.g., water capacitance, carbon sequestration, and both nutrient retention and cycling [17,20] and precision agricultural management. With some justification, early studies noted similarities between prairie potholes and forest-steppe zapadiny. However, before applying tools developed in North American research to Russian systems, there is a need for quantitative evaluation of concepts that emerged in earlier local studies.

One of the key hypotheses developed during the 1960s is that the major source of recharge for shallow groundwater in areas such as the Oka-Don Lowland of the Tambov region in European Russia is depression-focused infiltration during snowmelt [21]. In a very recent study an indirect method was used to calibrate the groundwater recharge to hydraulic conductivity ratio for application in an analytical steady-state solution of the 2D shallow groundwater flow equation using soil redoximorphic features of typical classified catenas of the Samovetc catchment in this lowland [22]. In the cited study, the same recharge rate was prescribed for all points along a topographical transect. In contrast, in the study presented here, the spatial variability of depression-focused groundwater recharge along the transect was studied in a field campaign in spring 2021 during, immediately after snowmelt, and several weeks later.

There is no single method for classifying remote-sensing data for the ponds’ retrieval. The methods and materials used vary greatly depending on the region of study, season of the year, image resolution or type of the pond. In terms of wavelengths used in the electromagnetic spectrum, they are visible (RGB), near infrared (NIR), shortwave infrared (SWIR) and thermal infrared (TIR) [23,24]. In addition to optical methods, data from RADAR and LIDAR are also used [25]. Methods for extraction of small water bodies are divided into four groups. The first group is the threshold methods, the essence of which is the discretization of individual spectral channels or spectral indicators based on expert or experimental threshold values [26,27]. The second group of methods are statistical methods, such as those using multivariate regression or discriminant analysis. Classification methods (the third group) are a matrix of combinations of different methods—this is a pixel or object-oriented approach, classifications with or without training, various classification machines; for example: a random forest or support vector machine, neural algorithms [28,29,30,31,32]. There are also various special techniques (group four) such as entropy-based computer vision techniques [33].

In this work, remote-sensing data were used to construct a mass balance and estimate volumes of ephemeral ponds by object-oriented supervised classification of high-resolution Planet Labs images of the Tambov area acquired from April to May 2021. The data acquired on dynamic changes in delineated ponds, in combination with a DEM, observations using an unmanned aerial vehicle (UAV), a widely accepted method for calculating evaporation, and visual hydrological observations were used to estimate infiltration volumes and rates through the depression bottoms and account for their spatial and temporal variability. Considering that groundwater recharge from the depressions’ bottom is very area-focused and occurs episodically during the snowmelt, the process is not usually accounted for in the regional-scale evaluation of the groundwater resources in Tambov region. To include the impacts of spatial heterogeneity and dynamic fluctuation the depression-focused infiltration may be modeled numerically [8]. To examine the early hypothesis [21] on a critical role of depressions in ground-water recharge in a forest-steppe region through the simplified approach for estimating recharge, this paper aims: (1) to determine the variations of the pond recession and infiltration rate in time and between the depressions due to systematic (vertically-varying hydraulic conductivity) and random factors (presence of clogging or frozen layers, pond- surface drainage network connection); (2) to determine the role of such spatial variations through numerical analysis of shallow groundwater model for the simplified 2D case; and (3) to determine a method for calculating the volume of recharge through depression in other catchments both with and without the requirement of numerical modeling and data assimilation. The result allowed identification of the volume of intercepted water during snowmelt and calculation of the rate of water recession and infiltration rates in closed depressions for the first time for the study region. Use of the horizontal variation in parameters obtained along the studied transect substantially improved results of the shallow ground water model developed in the cited study [22].

## 2. Materials and Methods

Remote-sensing data were used to construct a mass balance and estimate volumes of ephemeral ponds by object-oriented supervised classification of high-resolution Planet Labs images of the Tambov area acquired from April to May 2021. The data acquired on dynamic changes in delineated ponds, in combination with a DEM, observations using an unmanned aerial vehicle (UAV), a widely accepted method for calculating evaporation, and visual hydrological observations were used to estimate infiltration volumes and rates through the depression bottoms and account for their spatial and temporal variability. The acquired time series of changes in the volume of nine temporary ponds enabled parametrization with a negative exponential curve. A time series of the infiltration rate, calculated from the water balance, was used to estimate the total amount accumulated during the event, and both the initial (maximum) and saturated (minimum) infiltration rates per unit area. 

### 2.1. Study Area

**Figure 1:**
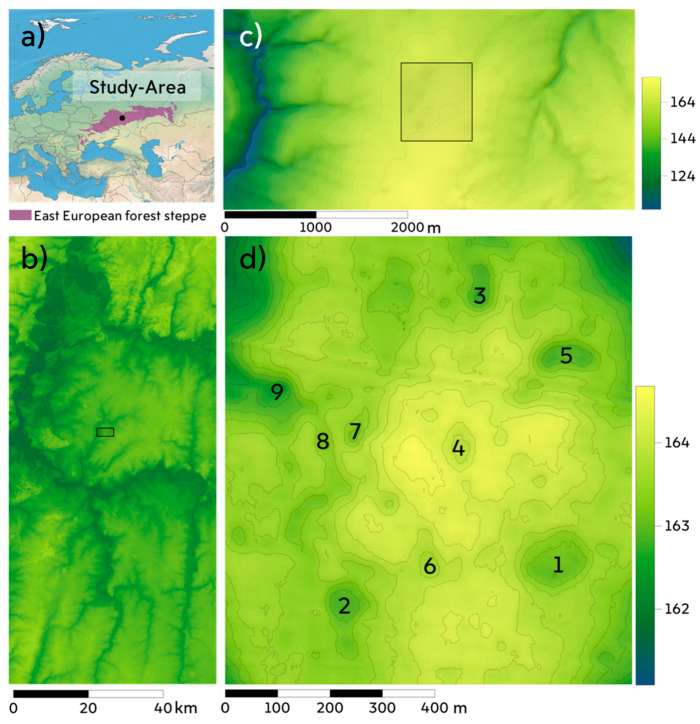
The study area. (**a**) Location of the study area in the forest-steppe biome of Eurasia. (**b**) Location of the study area in the catchment of the Matyr River-Oka-Don Lowland (SRTM). (**c**) Digital elevation model (DEM) of the interfluve of the Samovets brook; the studied territory of the depressions is marked with a rectangle. (**d**) Unmanned aerial vehicle (UAV) DEM of the study area, the numbers indicate numbers of closed depressions filled with pond water in the spring of 2021 (for details, see Table 2).

The study area covers approximately 560 ha in the center of the Oka-Don lowland (52°37′ N, 40°2′ E) in the Petrovsky district of the Tambov region, Russia (Figure 1). The lowland is the largest in the forest-steppe biome. With elevation ranging from 120 to 180 m above sea level, on average it is 100 m lower than adjacent territories. The lowland has a semi-arid climate with long winters, pronounced spring snowmelt events and relatively dry summers with an annual precipitation to potential evapotranspiration ratio of 0.8. According to data recorded at a meteorological station 10 km north of the study site, during the period 2005–2020 the annual temperature was 6.9 °C, and average monthly temperatures in January and July were −8.6 °C and 21.0 °C, respectively [34]. Mean annual precipitation during this period amounted to 550 mm (of which 113 mm fell during periods with sub-zero temperatures), and the mean snow height before onset of snowmelt was 320 mm, very similar to the recorded historical climatic norm for 1961–1990 (290 mm).

The soils are mainly chernozems and the area is mainly used for cultivating crops (typically wheat, corn, sunflower, soy, sugar beet), despite hindrance by water shortages. Clay and loamy deposits, generally 5–15 m (but sometimes up to 40 m) thick, with boulders of glacial origin, underlie a layer of loess-like loam with thickness ranging from 2 m in the lower parts of slopes to 30 m in the interfluve. The upper layer is porous and can both accumulate and retain moisture, while the glacial clays and loams form a local aquiclude for infiltrated surface waters. Shallow groundwater above this aquiclude is permanent and forms a continuous layer in the focal catchment. Evidence of stagnic condition in topsoil is restricted to the presence of albic material in the lower part of the humus horizon in grey gleysols in the depression bottom. There is clear evidence of gleyic conditions in the soil morphology (Fe-Mn concretions, Fe masses, pore lining, reduced matrix) in the catchment and continuous presence of water saturation below 2–3 m depth in the poorly drained soils and 1 m depth in the waterlogged soils. The latter was confirmed by a few cases of drilling in different years and seasons (the WTD is consistently highest after the springflood) and automated measurements in 2019 (a year with extremely low snow accumulation). Physical properties of the surface loams have contributed to development of closed depressions of multifactorial genesis, which are widely spread throughout the Oka-Don lowland. These depressions delay the runoff of surface waters into rivers [21] and transfer surface runoff to groundwater, thereby replenishing the groundwater and moistening the surrounding soil. The closed depressions are filled with water in the spring when the snow melts. Snow located in the catchment area of each basin melts and replenishes it, usually in mid-March to early April. At the end of spring, surface water only remains in small parts of the depressions, and in summer they usually dry up completely, in contrast to the closed depressions of the Pothole Prairie Region. Monitoring the dynamics of water volume and its filtration enables estimation of amounts of valuable additional moisture entering the soil in this semi-arid region.

### 2.2. Input Data

**Figure 2:**
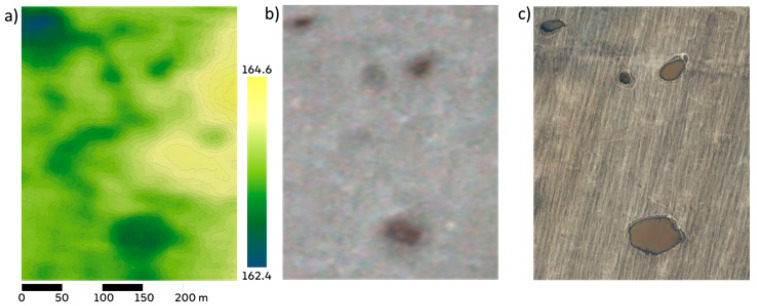
Sources of spatial data. (**a**) High-resolution DEM obtained photogrammetrically with colors indicating heights. (**b**) Planet Labs’ digital images of terrain in the visible spectrum with 3 m resolution. (**c**) High-resolution digital image obtained using the UAV in the visible spectral range.

Water dynamics in a closed depression in spring 2021 was tracked and modeled using the following three types of data (Figure 2): ultra-high resolution (25 cm) digital terrain and elevation models obtained using a small UAV—DJI Mavic 2 Pro, orthophotomaps of terrain in the visible range with a ultra-high resolution (25 cm) from UAV, orthophotomaps of high resolution (3 m) in the visible range from the sensors of the RapidEye and SkySat mini-satellites of the Planet Labs system [25]. Precipitation data were obtained from the nearest weather station with daily resolution. Evaporation data from the water surface was obtained using Penman’s equation and meteorological input from the same station.

In this study we used stereophotogrammetry, i.e., estimation of three-dimensional coordinates of points on an object from two or more photographic images taken from different positions by the small UAV. In this article, we used a standard method for constructing a digital terrain model using a small UAV with an accuracy of 0.03 m (hereafter, the UAV DEM).

For this, we used the Mavic 2 Pro routing app (DroneDeploy.com). Geolocation markers were located on the ground, and their positions were determined using the STONEX GNSS system (flight altitude, 150 m; image overlap, 75%). We processed the data using Agisoft Metashape and created a dense point cloud to generate a digital terrain model. We manually filtered points associated with agroforestry areas within the fields in ArcGis Pro using the field mask and the vegetation mask. The masks were obtained by manual decoding the UAV materials. Orthophoto maps generated from free satellite photos obtained via Bing were used to identify trees. Points related to heights of the trees were removed. A digital model of the territory with 25 cm resolution was created from the remaining point cloud using the kriging interpolation tool in ArcMap. An orthomosaic was created in Agisoft Metashape and exported with 25 cm resolution.

A time series of high-resolution visible orthomosaics (with 1–3 m resolution) at times when there was no cloud cover, from the beginning of spring snowmelt to the drying up of temporary water bodies in early summer were downloaded from Planet Labs Inc. Images of the scenes were downloaded when there was no cloud cover, from the beginning of spring snowmelt to the drying up of temporary water bodies in early summer.

#### 2.2.1. Delineation of Water Bodies

Orthophotomaps generated using an UAV allow correct interpretation of water surfaces, as they can be visually inspected to delineate water/dry surface boundaries accurately. Orthomosaic maps from Planet Labs have lower resolution and higher atmospheric noise. Therefore, we used the Interactive Supervised Classification tool in the ArcGIS Pro desktop application to delineate water bodies in them. For this we created a water feature training set from the ultra-high resolution orthomosaic, and used it to enable automatic recognition of water bodies in the Planet Labs orthomosaics via object-oriented supervised classification, as implemented in the ArcGis Pro raster classification tool [35]. It is well established that object-oriented classification is superior to pixel-based classification for high-resolution images [36], and it has been previously used to delineate similar depression-shaped natural systems [37,38].

**Figure 3:**
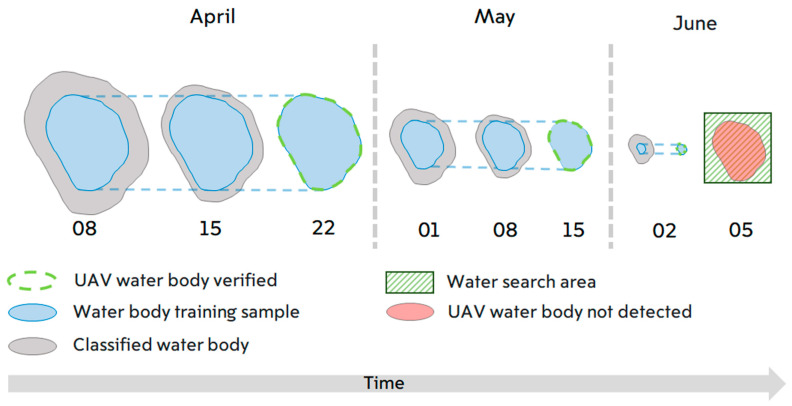
Schematic illustration of the water surface classification method. The arrow at the bottom indicates the general trend of depressions drying (from left to right) in spring. The green dashed lines around the blue areas show the extent of the water as photographed by the UAV on the control dates (22 April, 15 May and 2 June). The gray color indicates the water surface resulting from object-oriented image classification trained on a subset of points from the later control surveys by the UAV. Three time intervals were used for the training. The green shading at the end is the area searched for a water mirror surface by the UAV on a day when the pond had already disappeared (light red fill).

The classification involved the following steps. First, the analyzed raster layer was constrained by a field cadastral border [39] buffered 15 m on each side to prevent inclusion of objects rather than a bare soil surface without water (e.g., an agricultural field with no vegetation in early spring) and surfaces that may be flooded with water. Masking was applied to avoid possible classification errors by excluding unnecessary objects (trees, roads, buildings, etc.). The second step was imaging segmentation, based on a mean shift procedure, by criteria of the minimum segment size expressed in pixels [40,41] implemented in ArcGIS Pro, to merge adjacent pixels of relative homogeneity—preferentially based on spectral (color) characteristics—into image objects. Unitless segmentation scale parameters determining the average size of objects governing the degree of homogeneity allowed for pixel merging was set to 10 on the RGB scale. The third step was creation of a training set. As summer approaches, ponds in the depressions always shrink (Figure 3). Thus, water surfaces present on the date of a UAV flight were always present on the preceding dates, and three training samples were created for groups of dates before each UAV survey (Figure 3). Each training sample contained two categories: water and soil surface. Finally, the random forest (RF) method [42,43] and support vector machine (SVM) [44] for supervised classification of segmented images was applied, yielding a binary (water–not water) raster. The testing set from the next UAV survey was used to validate the resulting binary models. This enabled identification of the water surface areas in each period. However, in the classified images, the boundary of the ponds does not have a constant height relative to the DEM of the UAV. To avoid this unnatural variation of height the classified raster was transformed into a vector containing only water polygons. Along the outer boundary of the water polygon, the DEM values of the UAV were sampled with a frequency of 25 centimeters. The median was calculated from the extracted values. The contour was then drawn for the second time, now in accordance with the average value on the UAV DEM, thus, the outer boundaries of the ponds were forced to have a constant height value. The described procedure allowed us to avoid misclassification of the Planet Labs mixed pixels due to relatively low resolution as we operated with the vector area, not the raster area. For the comparison purely pixel-based classification was also made. The DEM and water polygons vector were used to calculate the volume of water in each depression on the days the images were taken. We used the Surface-Volume tool from ArcGIS Pro to calculate the area and volume between the surface and the reference plane (Polygon Volume (3D Analyst). This provided the water content in each depression in cubic meters on each of the days.

The maximum surface water area of a depression corresponds to the volume of water up to its overflow point, defined here as the minimum value of the height in the UAV DEM along its drainage basin vector boundary [45]. The watershed boundary was defined by the Basin tool in ArcGis based on the raster of the flow direction, derived from the UAV DEM using the “Direction of flow” tool in ArcGis Pro. The water layer (mm) in the catchment area of each depression required for its maximum volume is equal to its total maximum volume of water divided by its entire catchment area.

#### 2.2.2. Evaporation

Results of a previous comparison suggest that all of three conventional methods for estimating evapotranspiration from water-filled and vegetated depressions have acceptable applicability for estimating evaporation from open water [46]. The most convenient of these methods, the classical form of the Penman equation [47,48], was used in this study to estimate potential evaporation:

(1a) $$E_{PEN}=\frac{\Delta }{\Delta +\gamma }\;\frac{R_{n}}{\lambda }\;\frac{\gamma }{\gamma +\Delta }\;\frac{6.43E_{A}}{\lambda },$$

Here: *E_PEN_* is potential (open water) evaporation (mm/d); *R_n_* is net radiation at the surface (MJ/m^2^/d); Δ is the slope of the saturation vapor pressure curve (kPa/C); γ is a psychrometric coefficient (kPa/°C); λ is the latent heat of vaporization (MJ/kg); and *E_A_* is the drying power of the air, which can be found using the following Dalton-type formulation:

(1b) $$E_{A}=f\left(U\right)D=\left(1+0.536U\right)\left(e_{s}-e_{a}\right)$$

Here: *f*(*U*) is a wind function with linear coefficients for the original Penman equation (1948, 1963); u is the wind speed at 2 m height (m/s), *D* = ($e_{s}-e_{a}$) is vapor pressure deficit (kPa); *e_S_* is saturation vapor pressure (kPa); and *e_a_* is actual vapor pressure (kPa).

Open-water evaporation was computed from readily available data as previously described [49] and implemented in the Evaplib Python library [50]. Input data for this were air temperature (T, °C), solar radiation (RS, MJ/m^2^/d), relative humidity (RH, %), and wind velocity (u, m/s). 

In the absence of actinometric measurements of net radiation at the surface, this was calculated from amounts of cloud cover recorded at the weather station and a previous regional calibration [51].

(2a) $$R_{a}=\frac{24\left(60\right)}{\pi }G_{sc}d_{r}\left(\omega_{s}\sin\left(\phi \right)\sin\left(\delta \right)+\cos\left(\phi \right)\cos\left(\delta \right)\sin\left(\omega_{s}\right)\right)$$

Here: *R_a_* is extraterrestrial radiation (MJ/m^2^/day**), *G_sc_* is the solar constant (0.0820 MJ/m^2^/min), *d_r_* is the inverse of the relative distance between the Earth and Sun, *ω_s_* is the sunset hour angle (rad), *ϕ* is latitude (rad), and *δ* is solar declination (rad).

(2b) $$d_{r}=1+0.033\cos\left(\frac{2\pi }{365}J\right)$$

where *J* is the day of the year; $\delta =0.409\sin\left(\frac{2\pi }{365}J-1.39\right)$;

$$\omega_{s}=\arccos\left(-\tan\left(\phi \right)\tan\left(\delta \right)\right)\;;N=\frac{24}{\pi }\omega_{s}$$

Solar radiation, *R_s_*, can be calculated from the amount of cloud:

(3) $$R_{s}=\left(a_{s}+b_{s}\left(1-N\right)\right)R_{a}$$

where *N* is the amount of cloud (ranging from 0 for clear sky to 1 for full cloud cover), while *a_s_* and *b_s_* are Angstrom values, and without regional calibration values of 0.25 and 0.50, respectively, are recommended [52].

Net longwave radiation (*R_nl_*) can be estimated from the air temperature, actual vapor pressure, and solar radiation. Net longwave radiation is expressed by the Stefan–Boltzmann law:

(4) $$R_{nl}=\sigma \left(\frac{T}{max_{4_{min}^{4}}^{2}}\left(0.34-0.14\sqrt{e_{a}}\right)\left(1.35\frac{R_{s}}{R_{so}}-0.35\right)\right)$$

where *T_max_* is daily maximum air temperature (K), *T_min_* is daily minimum air temperature (K), and *R_so_* is clear-sky radiation (MJ/m^2^/day) according to:

(5) $$R_{so}=\left(a_{s}+b_{s}\right)R_{a}$$

We applied a constant albedo of 7% (0.07) for water surfaces in the calculations, based on the latitude and published mean reference values [51].

(6) $$R_{n}=\left(1-\alpha \right)R_{s}-R_{nl}$$

We calculated daily evaporation values. Input data for Equations (1a), (1b) and (4) and daily precipitation were obtained from the nearest meteorological station (at Lipetsk city).

#### 2.2.3. Water Balance and Groundwater Model Recalibration

Infiltration rates (mm/day) were calculated from the daily water balance equation:

(7) $$F=1000\cdot \left[-\Delta V-A_{t}\left(E_{PEN}-P\right)\right]/A_{t}$$

where −ΔV is the daily rate of reduction in pond volume (m^3^), *A_t_* is the current pond area (m^2^), and *P* is the daily precipitation (mm/d).

The volume of a pond on a given Julian day (*dayT*) was derived from the volume on the first Julian day in a series (*dayF*) and the following negative exponential equation:

(8) $$V=a\cdot e^{-c\cdot \left(dayT-dayF\right)}$$

**Figure 4:**
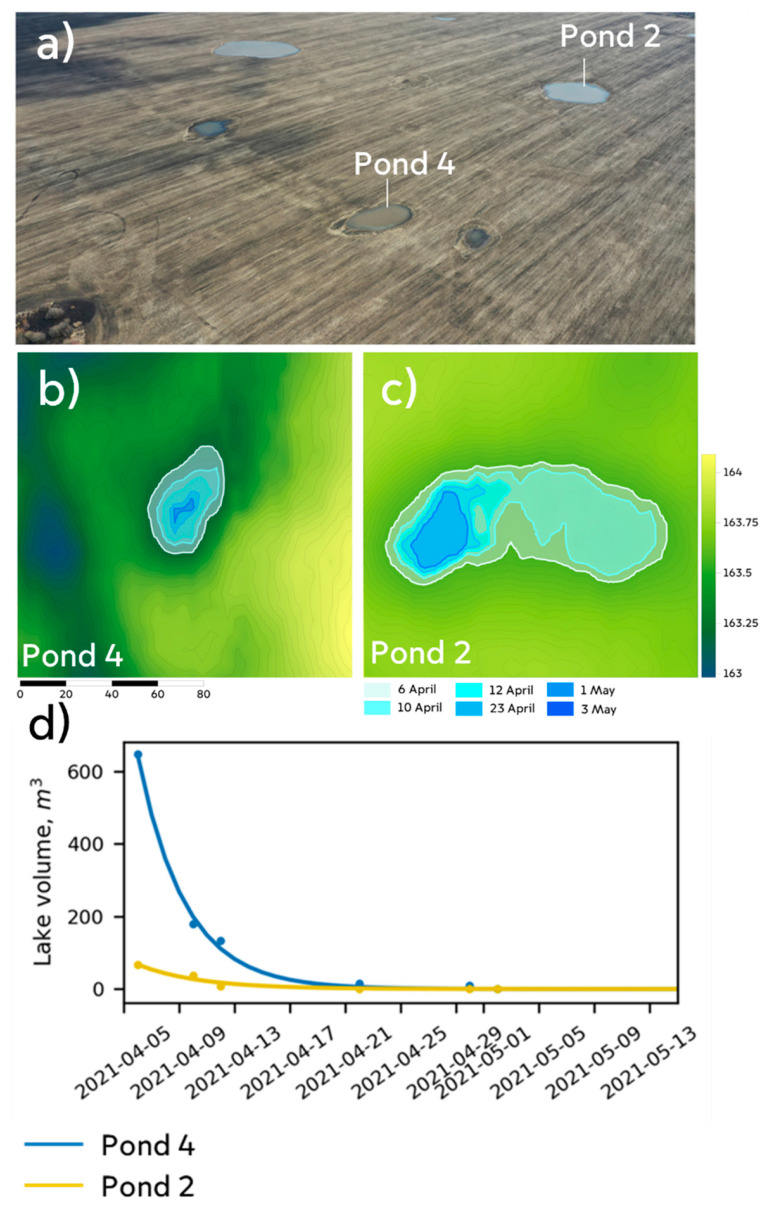
Temporal dynamics of selected ponds in the depressions. (**a**) Positions of Ponds 4 and 2 in a UAV photo image. (**b**,**c**) Groundwater levels in basins of the ponds on indicated dates based on the classification of images (highlighted in color by day). (**d**) Water volumes in the basins of ponds 4 and 2 on indicated dates (points) and negative exponential fits derived by Equation (8) (lines). Pond numbering as in Figure 1 and Table 2.

The scaling coefficient *a* and the power of the exponent *c* (the pond’s approximate initial volume and decay rate, respectively) were obtained by the least square method, which has given a fit with R^2^ > 0.9 for each of studied lakes (Table 2, Figure 4d). The estimated volume on each day was used to calculate the rate of reduction in pond volume in Equation (7) with a daily time step. 

A limitation of the method lies in the choice of the first day of infiltration, because it is impossible to determine the water boundary in depressions when they are covered with snow. Water infiltrates the soil when the temperature is already above 0 degrees Celsius, but classification of images with partial snow cover is problematic. Thus, the first Planet Labs image that was subjected to classification was the first when there was no snow cover according to the nearest (Lipetsk) weather station.

A 2D profile of the steady-state shallow water table depth was obtained by the analytical form of continuity equation with calibration based on soil redoximorphic features. In [22] the hypothesized relationship between archived morphological properties (redoximorphic features as indicators of gleyic conditions) of soils and a current hydrological process indicator (WTD) were established based on the expert knowledge of soil types, WTD co-occurrence, then verified under a hillslope flow continuity constraint expressed mathematically as a steady-state solution with two free parameters: hydraulic conductivity and recharge rate. Here the input horizontal transect of groundwater recharge rate was taken as a time integral of Equation (7). Spatially, it varied along the transect according to positions of the depressions in the landscape. Infiltration into the soil is not equal to the groundwater recharge rate, so relative values in the [0, 1] interval were used to describe the variability along the transect while the formal calibration of the absolute values of recharge rate to hydraulic conductivity (N/k) was preserved in the method. Time-averaged infiltration was calculated based on the volume of water that infiltrated in closed depressions. We established 10 regular topographic profiles representing the generalized transect, 3 km long and crossing the interfluve along the main slope with regularly (5 m) spaced points. At each standard point, the value of the water layer (mm) was extracted, which was filtered out in a closed depression. The 10 topographic lines were combined into a single profile by averaging values corresponding to the order of the points of the water layer. The regular placement of topographic profiles and sampling points was intended to optimize the two-dimensional characterization of additional moisture infiltration along the studied transect.

## 3. Results

The proposed combination of object-oriented image classification based on a time series of Planet Labs images and an orthomosaic derived from UAV surveys to verify the satellite data enabled highly accurate identification of the water mirrors of closed depressions during their drying (Cohen’s kappa = 0.99). Moreover, high-precision digital terrain models obtained using UAVs can be used to calculate volumes of water in closed depressions. 

We compared different methods of pond extraction for the scene on 9 April 2021, when the reference UAV image was obtained. Two supervised pixel-based classification methods were compared: random forest (RF) and support vector machines (SVM) providing results as a raster. Then the ponds boundaries were brought to a constant median value of the DEM to obtain vector pond polygons (also both from RF and SVM classification). Root mean square error (RMSE) and mean absolute percentage error (MAPE) of pond volume and area were the lowest for the vector approach and notably higher for raster approach (Table 1). SVM and RF errors were almost the same within the vector approach (Table 1), and it was decided to use RF as the most common in such studies. Contrary to [52], the novel way to vectorize the polygons based on idea of flat pond mirror with constant height brought a very notable increase in the quality of area and volume estimate. 

Results obtained using the described procedure show that the drainage process of the focal depressions follows an exponential equation (Figure 4, Table 2), with coefficients (Table 2) that presumably depend on various factors (e.g., the depressions’ source rocks and filtration areas), but we found no systematic quantitative relationships between the coefficients and considered parameters.

**Figure 5:**
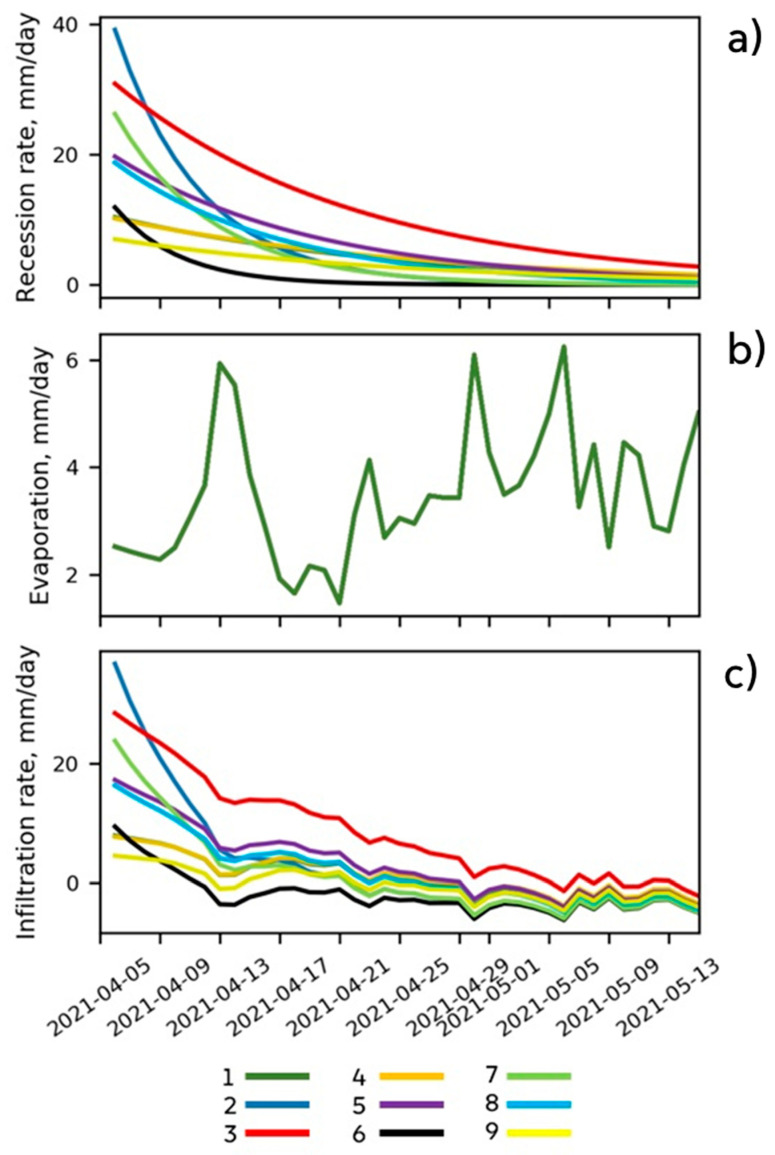
Rate of recession of the indicated ponds’ water levels (**a**), daily evaporation rate (**b**) and infiltration rates of the ponds estimated from the mass balance (bottom panel) expressed in mm of the water layer (**c**). The numbering of the ponds in the color legend follows Figure 1 and Table 2.

During the initial phase the rate of pond recession is much higher than later in the season (Figure 5 top). Notably less water is evaporated than infiltrates (Figure 5, middle and bottom), so the depression-focused replenishment of the groundwater is consistent with the previously mentioned hypothesis that the major source of recharge for shallow groundwater in the study area (and similar areas) is depression-focused infiltration during snowmelt [21]. There are two phases of infiltration—fast and slow (Figure 5, bottom). Measurements during the fast phase enable estimation of the unsaturated soil’s refill rate and capacity (Table 2). During the slow phase the change in infiltration rate from day to day is much smaller. The saturated hydraulic conductivity decreases strongly with depth under a depression [8], reflecting the effects of the decreasing frequency of fractures with depth, and the flow is presumably limited by the lowest layer with the smallest frequency. Thus, the infiltration rate estimated during the slow phase provides an approximation of the hydraulic conductivity (Table 2), corresponding to the maximum possible flux out of the soil column.

**Figure 6:**
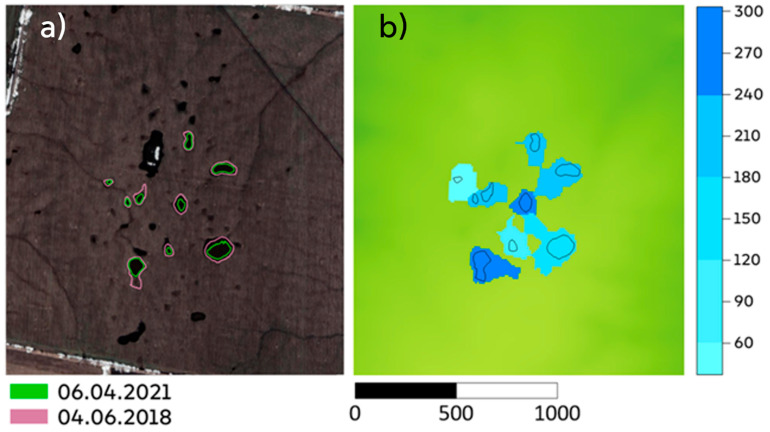
(**a**) Boundaries of the maximum potential filling of closed depressions (up to their overflow heights) shown in white in a Planet Labs image from 4 April 2018, with color shading and magenta contours, and the maximum pond boundaries in 2021 from 6 April 2021 (green contours). In 2018, the investigated closed depressions were overflowing. (**b**) Shades of blue indicating the layers of water (in mm) that must enter the depressions from their catchment areas to completely fill them.

Overflow can occur from any closed depression (Figure 6). The probability of spillage depends on multiple factors, including elevations of the lowest point in the catchment area and the depression’s overflow point. In 2018, water reached the overflow point in almost all the depressions considered here (Figure 6). Thus, the initial volumes (*a* coefficients) obtained for the nine studied ponds can be used in speculation regarding the effects of the landscape morphometry and meltwater input on initial volumes of ponds after snowmelt. 

The results also indicate that the hypothesis of a quantitative linear relationship between the volume of water accumulated in a depression and the catchment area of the basin is only partly correct. The water volumes do not appear to be linearly related to the depressions’ catchment, because the amount of water in a depression depends on the catchment area and maximum volume that can be stored in it. Excess water will flow through the overflow point without replenishing the water table. Limits of the possible volume and layer of water intercepted by the focal depressions, which limit their ability to converge surface runoff into underground flows, were identified. The maximal layer depends on the catchment area of the depression and height of its overflow (Figure 6, right).

The water layer filling closed depressions during snowmelt in the forest-steppe zone is highly dynamic. From 2005 to 2021, the snowmelt water layer (snow water equivalent, SWE), reconstructed from the statistically corrected snow height and snow density data series, varied from 50 to 300 mm. The derived snowmelt water layer during this period has a binomial distribution with two maxima, at 50 and 200 mm SWE. Analysis of the meteorological data showed that closed depressions did not overflow during snowmelt in 60% of cases, on average, from 2005 to 2021. This corroborates the finding that in most cases closed depressions intercept the surface runoff and transfer it to groundwater. Frequencies of overflow were lowest for Ponds 2 and 4 (around 10%) and highest for Pond 9 (90%).

**Figure 7:**
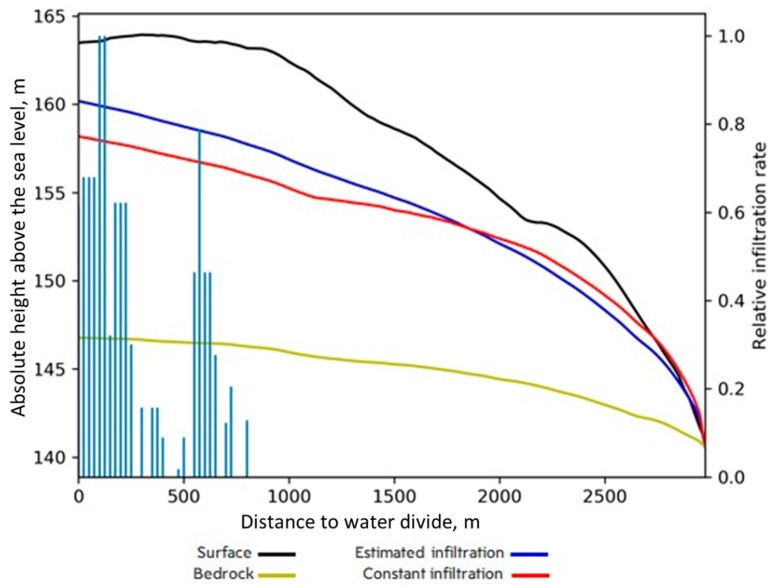
Cross-section of the catena with water table depth (WTD, blue and red lines) adjusted to correspond to soil group [22]. The yellow and black lines indicate the position of the bedrock and DEM profile, respectively. The blue and red lines respectively indicate WTD obtained with infiltration along the transects estimated from the depressions’ positions and specific infiltration rates (as indicated by the blue bars and right y axis) and constant infiltration along the transect.

Figure 7 illustrates the simulations of the shallow groundwater level for cases with the recharge rate either constant or spatially varied along the transect. The parameter N/k was restricted by the requirement for correspondence between the simulated WTD and range of WTD for soils of each type from expert knowledge (Table 2 in [22]) in distance intervals across the catena’s whole toposequence. For example, if very poorly drained soils (under depression bed) are present in M unit intervals, those in which WTD > 3 m (too deep) were counted with 0 weight and the others with 1 weight. The same procedure was then applied for each of the intervals with the other soil types, then the sums were added for all groups and scaled to the total number of unit intervals in the catena toposequence to obtain the accuracy in percent. Simulation of WTD was successful for the generalized transect in terms of correspondence between the simulated WTD and ranges of WTD obtained from the indirect soil indicators (redoximorphic features) and expert knowledge both in the cases of constant and spatially variable recharge. However, the required accuracy threshold was set at 97%, and was met for the spatially variable recharge. A significantly lower threshold (80%) was satisfied for the constant recharge case. Therefore, the method to estimate depression-focused infiltration proposed here can make the shape of the water table profile more realistic. 

## 4. Discussion

The word steppe is usually associated with the Russian plains, but the northern part of this ecoregion has notable similarity to North American prairies. The lithological and geomorphological similarity of the Tambov region to the Saskatchewan and Alberta provinces in Canada enables direct comparison of the depression-focused infiltration into their soils through temporal ponds that are very similar in size distribution and shape. The recession rate of the ponds after snowmelt obtained in this study is similar to that derived from an artificial flooding experiment in the C24 depression, northwest of Calgary, Alberta, Canada, in 2004 [8]. As in the cited study [8] and another previous investigation [4], we found that evaporation accounts for a much smaller proportion of the pond water balance loss term than infiltration into the soil. The pre-event pore space available for filling with infiltration water was not directly measured in this study. However, data from a depression monitoring site in the study region in the years 2003–2005 show a spread of 30–400 mm of water deficit to saturation. An assumption underlying our two-stage infiltration conceptual model is that pores of the soils below the bottom of a pond are all filled to saturation during the first stage down to the shallow groundwater depth (approximately 2 m). Thus, the inflow is restricted by the bottleneck hydraulic conductivity below this point, which is an order of magnitude lower than in the upper soil layers [8] and also by the gradual rise of the water table when two fronts of water are jointing. The soil refill amount of 34 to 172 mm recorded in Table 2 fits well into this range. There is also similarity with the refill amount (148 mm) obtained in the cited Canadian study [8]. A strength of our study is that the infiltration rate was estimated for nine ponds, not just one pond such as the well-studied experimental pond C24. The variation (four-fold) in infiltration between those ponds (Table 2) could not be explained by the pond size or topographical settings. Thus, it is not sufficient to apply infiltration data from one pond to other ponds as this leads to large errors. The differences are likely due to diverse factors, inter alia physical properties of the soil associated with their lithological and textural characteristics, the thawing rate and ice content, abundance of root channels and other pathways for preferential flow. We conclude that there is no straightforward analytical way to characterize this spatial variability, but use of data obtained by the methods proposed here in conjunction with appropriate hydrological models and high-resolution satellite images is highly promising.

Here, we used the steady-state continuity equation in kinematic wave form parameterized using expert knowledge of the links between typical water table depth (WTD) and redoximorphic features of soils with different hydromorphy degrees [22]. In this simulation, we were able to account for variation in infiltration rates in the catena using real data on depressions’ positions within the transect. However, calibration was still necessary because the infiltration and recharge are split in time by unsaturated zone processes. In future research, we plan to develop a model conceptually similar to the VSMB Depression-Upland System (VSMB-DUS) model [8] using data acquired in investigations of the surface water–groundwater interaction in individual depressions and their catchments. The planned model will be based on the watershed hydrological WASA-SED model [53], which already discretizes focal watersheds into hierarchical levels (sub basins, land units, terrestrial components, soil-vegetation components). Land units are representative catenas and terrestrial components can be easily supplemented with depressions and uplands providing surface flow to them by an already incorporated horizontal flow mechanism. For the terrestrial components prescribed as depressions, the temporally varying fluxes obtained by the method developed here will be used as upper boundary conditions. Collection of field data is planned to obtain saturated hydraulic conductivity values for the vertical levels besides the bottom soil layer. Groundwater depth measurements will provide calibration for the drainage rates from the deepest soil layer and validation for the dynamic version of the WASA-SED shallow groundwater flow sub-model. In this manner, groundwater recharge will fully account for the spatial variability of depression density, such as prevailing areas of numerous depressions at the water divide. 

In this study, we derived the saturated hydraulic conductivity, not for a single point, but aggregated for the area of depressions. Most grid data used represent points, but landscape-level data are essential inputs for a hydrological model. A hypothesis under test is that soil hydraulic properties are related to landscape position and topography [54]. If so, elucidation of these relationships could greatly enhance pedotransfer functions for estimating saturated hydraulic conductivities at the level of land units and terrestrial components, not just points. Our study, based on remote sensing, provides an example of such derivation because the hydraulic conductivity is based on the depressions’ water balance accounting for their positions in the landscape. 

A limitation of this study lies in the assumption that all snowmelt runoff from the upland was routed to the depressions before the initial day of the study, and water volume within each depression exceeding its maximum storage capacity overflowed directly into surface runoff with no contribution to infiltration into the soil. However, it is widely acknowledged that depressions tend to form fill-spill networks, where overflow from one depression feeds an adjacent depression [5,6]. This process can be modeled [6,10,15,16,17], but studies of fill-spill processes have primarily focused on effects of depression storage on surface flow to streams rather than depression-focused groundwater recharge. Visual observations during the hydrological phase after the most active snowmelt showed no signs of connectivity between depressions at our study site, but that was typical for the active snowmelt phase itself of about a week duration. We justify the restriction of our approach with the hypothesis that non-stationary volumes of the depression ponds when snow is still present contribute little to total infiltration, partly due to the frozen state of the soil. 

## 5. Conclusions

Estimation of infiltration through ponds is an important step toward the challenging goal to estimate depression-focused recharge of groundwater, and thus evaluate this important resource, in the forest-steppe zone of Russia. Using high-resolution Planet Labs images and widely evaluated tools for object-based image recognition, we have developed a relatively simple method to reconstruct a time series of infiltration into the soil under ponds and estimate landscape-scale saturated hydraulic conductivity. The simulation of the steady-state groundwater profile for the topographical transect fed with data on relative water supplies through depressions along the transect was more consistent with observations (based on soil redoximorphic indicators of water level) than the simulation fed with a uniform recharge function. Further development is needed to assimilate the data generated with consideration of the spatial variability of pond infiltration into a process-based model of groundwater recharge that accounts for interactions between depressions and their catchments.

## Figures and Tables

**Table 1:** Root mean square error (RMSE) and mean absolute percentage error (MAPE) of the area and volume with true value taken from ultra-high resolution UAV estimate of ponds’ boundaries. Random forest (RF) and support vector machine (SVM) methods are compared for the pixel-based (raster) and median DEM height-based (vector) delineation of the image taken on 9 April 2021.

	Errors of Area Estimate		Errors of Volume Estimate	
	RMSE, m^2^	MAPE, %	RMSE, m^3^	MAPE, %
RF vector	7.8	1.4	2.5	5.9
RF raster	43.2	3.1	16.0	21.9
SVM vector	8.7	1.6	2.1	5.1
SVM raster	44.9	3.6	16.3	22.2

**Table 2:** Derived characteristics of the ponds during the decreasing volume phase after snowmelt from the maximum (starting day) to zero (final day). Coefficients a and c are from Equation (8) and R^2^ is the coefficient of determination for the negative exponential fit of the lake volume by the least square method.

Pond No. ^1.^	Maximum Volume, m^3^	Maximum Area, m^2^	Coefficient *a*	Coefficient *c*	R^2^	Total Infiltration, m^3^	Initial Infiltration Rate, m^3^/d	Soil Refill Capacity, mm/dd ^2^	ksat mm/d	Total Evaporation, m^3^	Total Precipitation, m^3^
1	640	6123	604	0.11	0.91	475	47	54	3	320	155
2	647	4275	645	0.29	1.00	594	153	163	3	113	59
3	173	1756	173	0.38	0.98	167	50	172	12	18	12
4	66	1283	67	0.23	0.95	56	10	53	4	24	14
5	416	3546	428	0.19	0.94	366	64	103	6	103	54
6	16	540	16	0.53	1.00	14	5	37	1	6	4
7	103	978	104	0.28	1.00	92	23	113	2	24	13
8	36	543	37	0.34	0.97	34	9	93	4	7	5
9	12	295	13	0.19	0.99	9	1	34	2	7	4

^1^ The numbering follows Figure 1. ^2^ dd—melt water peak event duration (days).

## Data Availability

The data presented in this study are openly available in Fil, Pavel; Yurova, Alla (2021), “Water bodies recognition for depression-focused recharge. Tambov region, Russia”, Mendeley Data, V1, doi:10.17632/pn3gdzhdy4.1.
